# Effect of risperidone on proliferation and apoptosis of MC3T3-E1 cells

**DOI:** 10.1590/1414-431X20188098

**Published:** 2019-02-25

**Authors:** Lei Zheng, Lixia Yang, Xin Zhao, Niya Long, Peifan Li, Yiming Wang

**Affiliations:** 1 Department of Mental Health and Psychiatry, First Affiliated Hospital of Soochow University, Suzhou, Jiangsu, China; 2 Department of Psychiatry, Affiliated Hospital of Guizhou Medical University, Guiyang, Guizhou, China; 3 The Sixth People's Hospital of Guiyang, Guiyang, Guizhou, China; 4 Guizhou Medical University, Guiyang, Guizhou, China

**Keywords:** Risperidone, MC3T3-E1 cells, Collagen 1, Tumor necrosis factor-α, Receptor activator of nuclear factor-κB ligand

## Abstract

This aim of this study was to assess the molecular mechanism of osteoporosis in schizophrenia patients with risperidone use. Here, we investigated the effects of risperidone on cellular proliferation and apoptosis of a preosteoblast cell line, MC3T3-E1. Cell viability and apoptotic rate of MC3T3-E1 were detected by cell counting kit-8 and flow cytometry at a serial dose of risperidone and at different time points, respectively. Bone transformation relevant gene serum osteocalcin (BGP), collagen 1, tumor necrosis factor-α (TNF-α), osteoprotegerin (OPG), and receptor activator of nuclear factor-κB ligand (RANKL) mRNA levels were determined by real-time PCR (qPCR). Their protein expression patterns were evaluated using western blot. The results revealed that risperidone dramatically inhibited MC3T3-E1 cell proliferation in a dose-dependent manner. It also significantly induced MC3T3-E1 cell apoptosis. TNF-α gene and protein levels were greatly enhanced after risperidone treatment. In contrast, BGP, collagen 1, OPG, and RANKL gene and protein levels were markedly downregulated. Our study indicated that risperidone suppressed MC3T3-E1 cell proliferation and induced apoptosis. It also regulated BGP gene and protein expression.

## Introduction

Schizophrenia is one of the most common mental disorders characterized by severe abnormal behavior and chronic relapsing (1). Patients take drugs for controlling symptoms for long terms. Currently, risperidone is one of the atypical antipsychotics for the most severe schizophrenia patients because of its excellent effects. However, studies show that patients receiving risperidone for a long time have increased risk of osteoporosis (2,3). The rate of femoral neck fracture in patients taking risperidone is more than two-fold that of the normal population, and it may cause the death of patients (4,5). The mechanism of osteoporosis in schizophrenia patients taking risperidone is unclear. Therefore, elucidating this mechanism may enhance the drug's safety and patient life quality.

Risperidone targets dopamine D2 receptor in the pituitary gland or 5-hydroxytryptamine receptor 2 (5-HT2 receptor) in the neurons of the hypothalamus (6,7). Its binding to the D2 receptors results in the release of prolactin and subsequently leads to hyperprolactinemia. A study indicated that the bone mineral density (BMD) of hyperprolactinemic women is 15–30% less than the normal population (8). Graham et al. (9) reported that hyperprolactinemia in humans and animals affects bone marrow cell metabolism and decreases BMD (10,11), increasing the risk of fracture. During bone formation, osteoblasts (12) play a critical role in the metabolism and remodeling of bone because these cells secrete rich bone metabolic relevant factors including osteocalcin (BGP) (13), type I collagen (collagen 1), tumor necrosis activator factor-a (TNF- α), osteoprotegerin (OPG), and receptor of nuclear factor kappa B ligand (RANKL). These biochemical changes in bone markers reflect bone functions.

Here, we cultured preosteoblast cell line MC3T3-E1 *in vitro*, and observed cellular proliferation and apoptosis after risperidone administration. At the same time, we detected the gene and protein levels of bone formation relevant biochemical markers BGP, collagen I, TNF-α, OPG, and RANKL. This study explored the effects of risperidone on bone formation and differentiation. It also provided the foundation for eliminating the side effects of risperidone.

## Material and Methods

### Cell culture and reagents

MC3T3-E1 subclone 14 was purchased from ATCC and cultured in Dulbecco's modified Eagle's medium (DMEM, Gibco, USA) supplement with 10% fatal bovine serum (FBS, Gibco), and 1% penicillin-streptomycin antibiotics. When cells were confluent to 80–90%, serial passage was performed. Cells were washed twice with D-Hank's solution and digested with 1 mL of 0.25% trypsin-EDTA (Gibco). Next, 10× culture medium was added for stopping digestion and a few T25 flasks were used for reseeding. Risperidone was purchased from Sigma (USA) (Cat #R3030). Later, 10 mM stock was prepared in dimethyl sulfoxide (DMSO) solution.

### Cell counting kit-8 (CCK-8) assay for cell proliferation and drug toxicity

To assess the effects of risperidone on MC3T3-E1 cell proliferation, CCK-8 kit was used to detect cell proliferation rate in an empty group (only medium, no cells), control group (with medium and cells, but no risperidone), and experimental group (with medium, cells, and different doses of risperidone). Briefly, 2×10^3^ MC3T3-E1 cells per well were seeded in a 96-well plate and incubated for 24 hat 37°C in 5% CO_2_. When cells were 80-90% confluent, the culture medium was replaced with fresh medium with no serum. Subsequently, 10, 50, 100, and 200 μmol/L risperidone was added to the medium in duplicate wells. Cells were cultured for 24, 48, and 72 h. CCK-8 reagents were added into 96-well plates and incubated for 4 h. Absorbance (AB) was measured at 450 nm wavelength. Cell viability (%) = [AB of experimental group – AB of empty group] / [AB of control group – AB of empty group] × 100%.

### Determination of apoptosis by flow cytometry

To evaluate the effects of risperidone on preosteoblast cell apoptosis, MC3T3-E1 cells were cultured in 12-well plates and treated with 50 and 100 μmol/L risperidone for 48 h. The control group was not treated with risperidone. Each concentration was set in triple wells. For cell apoptosis analysis, risperidone-treated and untreated cells were washed twice with cold PBS solution. Then, cells were harvested and stained with propidium iodide and FITC conjugated annexin V (BD Pharmingen, USA) following the manufacturer's instructions. All samples were analyzed in 1 h with a Canton analyzer machine (BD Pharmingen). Early and middle-late apoptotic rates were recorded. Data were analyzed using FlowJo software (USA). The results were generated from three independent experiments.

### Expression of bone development genes

To investigate expressions of genes specific for preosteoblast development, MC3T3-E1 cells were cultured with 50 and 100 μmol/L risperidone as the experimental group or without risperidone administration as the control group for 48 h. Cells were harvested by 0.25% trypsin-EDTA detachment and washed twice with phosphate-buffered saline (PBS) solution. Total RNA was extracted from cultured cells with 1 mL TRIZol reagent (Invitrogen, USA). Detailed protocol was followed according to the manufacturer's instructions. Total RNA was quantified by UV spectrophotometry at A260/A280. cDNA was synthesized after incubation at 55°C for 30 min, 85°C for 5 min, and 4°C for 10 min in the presence of reverse transcriptase. qPCR was run on ABI Step One Plus machine (Thermo Fisher Scientific, USA), with the following cycle conditions: 50°C for 2 min, 95°C for 10 min, 95°C 15 s, and 60°C for 1 min (40 cycles). BGP, collagen 1, OPG, RANKL, and TNF-α primer sequences for PCR are shown in Table 1. All gene expression levels were normalized by β-actin gene expression.

**Table 1:** Primers used for real-time reverse transcript-polymerase chain reaction.

Gene	Primer sequences (5–3)	Primer sequences (bp)	GenBank
Osteocalcin	Forward: CCCTGCTTGTGACGAGCTAT	90	NM_007541.3
	Reverse: GGGCAGCACAGGTCCTAAAT		
Collagen 1	Forward: CAATGGTGAGACGTGGAAAC	107	XM_021175426.1
	Reverse: GTTGGGACAGTCCAGTTCT		
OPG	Forward: AGGGCATACTTCCTGTTGCC	121	NM_008764.3
	Reverse: TGTTCATTGTGGTCCTCGGG		
RANKL	Forward: GGAAGCGTACCTACAGACTATC	132	NM_011613.3
	Reverse: AAAGTGGAATTCAGAATTGCCC		
TNF-α	Forward: CTAGCCAGGAGGGAGAACAG	149	NM_001278601.1
	Reverse: GCTTTCTGTGCTCATGGTGT		
β-actin	Forward: GTGCTATGTTGCTCTAGACTTCG	174	NM_007393.5
	Reverse: ATGCCACAGGATTCCATACC		

### Levels of preosteoblast development proteins

MC3T3-E1 cells with or without risperidone treatment were washed twice with cold PBS solution. Five hundred microliters of RIPA buffer containing 1× Complete^TM^ protease inhibitors (Roche, Switzerland) was added directly onto cell culture plates and incubated for 10 min at 4°C. Cells were collected in a new 1.5 mL Eppendorf tube. Then, samples were centrifuged at 16,128 *g* for 10 min at 4°C and the supernatant was separately transferred to new tubes. Sample concentration was determined by BCA protein assay (ThermoFisher Scientific). Samples were boiled for 5 min with 4× sample loading buffer and stored at –80°C. For western blotting, an equal amount of the sample was loaded onto 4–12% SDS-polyacrylamide gels together with molecular weight markers (Invitrogen) and transferred to nitrocellulose (NC) membrane. The blots were performed with primary antibodies in TBS containing 0.05% Tween-20 overnight and washed extensively. Secondary antibody conjugated-horseradish peroxidase (HRP) was incubated with NC membrane at room temperature for 1 h. Subsequently, the blots were visualized using the ECL kit (Roche), according to the manufacturer's instructions.

### Statistical analysis

All data are reported as means±SD. SPSS22.0 and GraphPad Prism 5 softwares (USA) were used to analyze the data. Statistical significance was determined by one-way ANOVA with significance levels of P<0.05.

## Results

### Risperidone inhibited MC3T3-E1 cell proliferation in a dose-dependent manner

**Figure 1:**
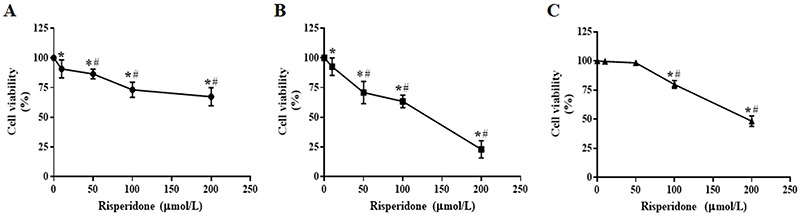
MC3T3-E1 cell viability after risperidone treatment. Viability curve at 24 (**A**), 48 (**B**), and 72 h (**C**) after administration of different concentrations of risperidone. Data are reported as means±SD, from three independent experiments. *P<0.05, comparing different doses of risperidone-treated group with the control group. ^#^P<0.05, comparison of present dose with previous dose of treated group (one-way ANOVA).

To explore the effects of risperidone on preosteoblast cell proliferation, MT3T3-E1 cells were cultured with variable doses of risperidone at different time points. Cell viability significantly decreased with increase in risperidone dose. In contrast to 24-h (Figure 1A) and 72-h (Figure 1C) culture, the maximal suppressing effects were achieved at 48-h (Figure 1B) culture. This experiment revealed that risperidone suppressed preosteoblast cell proliferation in a dose-dependent manner.

### Risperidone induced MC3T3-E1 cell apoptosis in a dose-dependent manner

**Figure 2:**
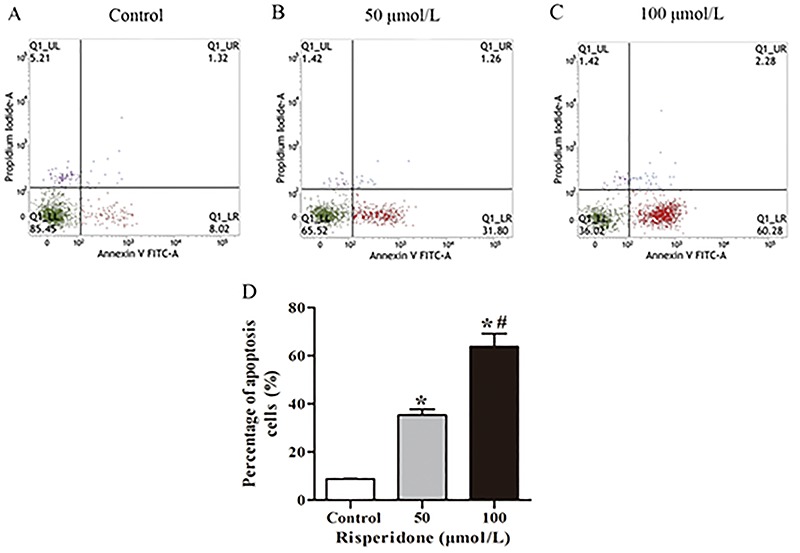
Risperidone reduced MC3T3-E1 cell apoptosis. The images indicate results for control (**A**), 50 μmol/L (**B**), and 100 μmol/L (**C**) risperidone. The graph in (**D**) was generated from three independent experiments. Data are reported as means±SD. *P<0.05, comparing 50 μmol/L or 100 μmol/L risperidone-treated groups with the control group. ^#^P<0.05, comparing 50 μmol/L treated group with 100 μmol/L treated group (one-way ANOVA).

Next, we detected the effects of risperidone treatment on preosteoblast apoptosis. Compared with the control group (Figure 2A), the apoptosis rate (FITC conjugated annexin V + Q1-LR gating region + Q1-UR gating region) of cells exposed to 50 μmol/L (Figure 2B) and 100 μmol/L (Figure 2C) of risperidone was significantly higher. Moreover, the apoptotic rate of 100 μmol/L risperidone-treated cells was markedly higher than that of 50 μmol/L-treated cells (Figure 2D, P<0.05). These data demonstrated that risperidone can cause MC3T3-E1 cell apoptosis in a dose-dependent manner.

### Effects of risperidone on expression of specific genes of preosteoblast development

**Figure 3:**
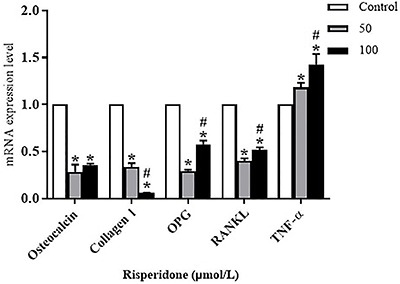
Osteoblast specific gene expression detected by RT-PCR. Osteocalcin, collagen 1, osteoprotegerin (OPG), receptor activator of nuclear factor kappa B ligand (RANKL), and tumor necrosis factor-α (TNF-α) gene levels in MC3T3-E1 cells were measured by RT-PCR in the presence of different concentrations of risperidone. All gene expression levels were normalized by β-actin gene expression. Data are reported as means±SD.*P<0.05, compared to the control group. ^#^P<0.05 compared to 50 μmol/L treated group (one-way ANOVA).

To explore the role of risperidone in preosteoblast development, gene expression of BGP, collagen 1, OPG, RANKL, and TNF-α were detected by qPCR method. Compared with the control group, expressions of BGP, collagen 1, OPG, and RANKL genes in risperidone-treated MC3T3 cells were downregulated (Figure 3). However, the TNF-α level was higher in risperidone-treated cells compared to the untreated group (P<0.05, Figure 3). Interestingly, expression levels of collagen 1 in MC3T3-E1 cells treated with 100 μmol/L risperidone were further downregulated compared to MC3T3-E1 cells treated with 50 μmol/L risperidone. On the contrary, OPG, RANKL, and TNF-α expressions were upregulated after administration of a higher concentration of risperidone.

### BGP, collagen 1, OPG, RANKL, and TNF-α protein expression in MC3T3-E1 cells

**Figure 4:**
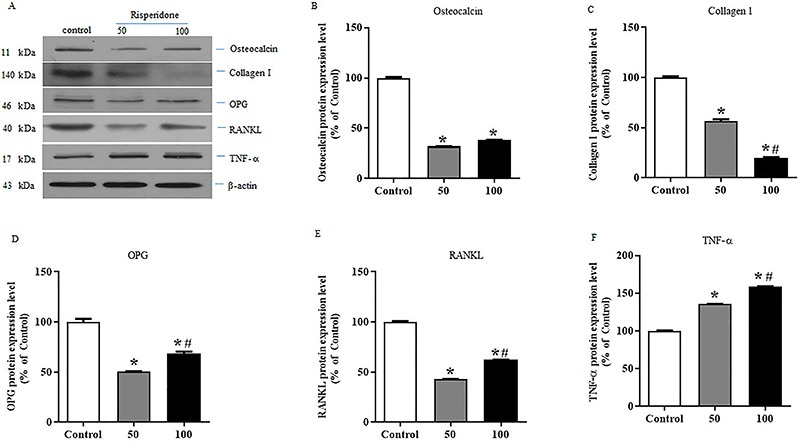
A, Osteoblast specific protein expression detected by western blot. Osteocalcin, collagen 1, osteoprotegerin (OPG), receptor activator of nuclear factor kappa B ligand (RANKL), and tumor necrosis factor-α (TNF-α) protein levels in MC3T3-E1 cells were measured by western blot in the presence of different doses of risperidone. **B**–**F,** Western blot density of the proteins normalized with β-actin. Data are reported as means±SD. *P<0.05 compared to the control group. ^#^P<0.05 compared to 50 μmol/L (one-way ANOVA).

Consistent with gene expression data from the qPCR method, protein levels of BGP, collagen 1, OPG, and RANKL declined in risperidone-treated MC3T3-E1 cells (Figure 4 A–E). In contrast, BGP protein level did not markedly change between 50 to 100 μmol/L risperidone-treated cells. TNF-α protein level was greatly enhanced after risperidone treatment (Figure 4F, P<0.05). These results confirmed that risperidone inhibited gene and protein levels of BGP, collagen 1, OPG, and RANKL. On the contrary, TNF-α gene and protein levels were enhanced after risperidone administration.

## Discussion

Risperidone is a widely used atypical antipsychotic medicine to treat schizophrenia, bipolar disorder, and autism (14). Common side effects include movement problems, sleep disturbances, constipation, and increased weight (15). However, recent data indicated that risperidone causes osteoporosis in the schizophrenia patients after long-term use (9,12,16). Here, we showed that risperidone inhibited preosteoblast proliferation and induced its apoptosis. Risperidone decreased BGP, collagen 1, OPG, and RANKL gene and protein levels, and increased TNF-α gene and protein levels in a dose-dependent manner. This revealed a novel mechanism of osteoporosis generation in patients taking risperidone.

Bone formation is actively balanced via interaction between osteoblast and osteoclast (17). This process is regulated by many factors including bone morphogenetic proteins, steroid hormones, and protein hormones (18–21). Our results revealed that risperidone inhibited MC3T3-E1 cell proliferation and induced apoptosis in a dose-dependent manner. We also found that risperidone upregulated TNF-α and downregulated the expression of other relevant proteins. TNF-α is a cytokine with multiple biological effects. It is secreted from osteoclasts and osteoblasts (22,23). Its level is high during aging, osteoporosis, and chronic inflammation (24). The roles of TNF-α on bone metabolism are mediated via the effects of proliferation, differentiation, and maturation of osteoblasts and osteoclasts. It plays a key role in osteoblast genesis and bone resorption process (25). The experiment showed that the molecular mechanism of osteoblast inhibition by TNF-α is via activation of NF-κB, which decreases phosphorylated Smad1 in bone morphogenetic protein 2 (BMP-2) signaling pathway (26). Dong et al. (27) and Jilka et al. (28) found that TNF-α promoted osteoblast apoptosis *in vitro*, a process involved in the pathology of osteoporosis. Therefore, we speculated that risperidone upregulated TNF-α gene and protein expression, which suppressed MC3T3 cell proliferation and induced osteoblast apoptosis. This finding revealed a major mechanism of osteoporosis in schizophrenia patients treated with risperidone.

Osteoblasts also secrete non-collagen proteins, including BGP and alkaline phosphatase to promote mineral deposition (29). It is well known that BGP is directly related to osteogenesis and mineralization rate (30). Our data indicated that gene and protein levels of collagen 1 and BGP in risperidone-treated MC3T3-E1 cells significantly declined compared to the control group. This corroborated previous reports. Overall, risperidone gave rise to osteoporosis by up-regulating TNF-α, and inhibiting collagen and BGP synthesis. Previous studies have also suggested that risperidone could trigger inflammatory and apoptotic response in macrophages cells (31,32). OPG/RANKL/RANK signaling pathway plays a critical role in the interaction between osteoblasts and osteoclasts (33). RANKL binds to OPG and belongs to the TNF receptor family (34,35). Its main function is promoting osteoclast differentiation, maturation, and activity. OPG is secreted from osteoblast and stromal cells. OPG and RANK competitively bind to RANKL. During this process, OPG suppresses osteoclast activity and maturation, which induces osteoclast apoptosis (13). OPG also inhibits apoptosis of TNF-α-induced osteoblasts (36). Our current study demonstrated that risperidone decreased the gene and protein expression of OPG and RANKL, which led to dysfunction of the OPG/RANKL/RANK signaling pathway and affected the differentiation of osteoclasts.

In conclusion, risperidone, an atypical antipsychotic medicine, enhanced TNF-α and deceased collagen 1 and BGP levels in MC3T3-E1 cells, which inhibited preosteoblast cell proliferation and induced its apoptosis. In addition, risperidone downregulated OPG and RANKL levels and affected the differentiation of osteoblasts and osteoclasts, which could lead to osteoporosis. This study of the mechanisms revealed a new strategy for the prevention of osteoporosis.
